# Detecting Incident Delirium within Routinely Collected Inpatient Rehabilitation Data: Validation of a Chart-Based Method

**DOI:** 10.3390/neurolint13040067

**Published:** 2021-12-09

**Authors:** Marco G. Ceppi, Marlene S. Rauch, Peter S. Sándor, Andreas R. Gantenbein, Shyam Krishnakumar, Monika Albert, Christoph R. Meier

**Affiliations:** 1 Basel Pharmacoepidemiology Unit, Department of Pharmaceutical Sciences, Division of Clinical Pharmacy and Epidemiology, University of Basel, 4031 Basel, Switzerland; marcogiacomo.ceppi@usb.ch (M.G.C.); marlenesusanne.rauch@usb.ch (M.S.R.); 2 Neurorehabilitation and Research Department, ZURZACH Care, 5330 Bad Zurzach, Switzerland; peter.sandor@zurzachcare.ch (P.S.S.); andreas.gantenbein@zurzachcare.ch (A.R.G.); shyam.krishnakumar@ksa.ch (S.K.); monika.albert@zurzachcare.ch (M.A.); 3 Hospital Pharmacy, University Hospital Basel, 4031 Basel, Switzerland; 4 Department of Neurology, University Hospital Zurich, 8091 Zurich, Switzerland; 5 Boston Collaborative Drug Surveillance Program, Lexington, MA 02421, USA

**Keywords:** neurocognitive disorders, delirium, patient-generated health data, rehabilitation, validation study

## Abstract

Background: Delirium is a brain condition associated with poor outcomes in rehabilitation. It is therefore important to assess delirium incidence in rehabilitation. Purpose: To develop and validate a chart-based method to identify incident delirium episodes within the electronic database of a Swiss rehabilitation clinic, and to identify a study population of validated incident delirium episodes for further research purposes. Design: Retrospective validation study. Settings: Routinely collected inpatient clinical data from ZURZACH Care. Participants: All patients undergoing rehabilitation at ZURZACH Care, Rehaklinik Bad Zurzach between 2015 and 2018 were included. Methods: Within the study population, we identified all rehabilitation stays for which ≥2 delirium-predictive key words (common terms used to describe delirious patients) were recorded in the medical charts. We excluded all prevalent delirium episodes and defined the remaining episodes to be potentially incident. At least two physicians independently confirmed or refuted each potential incident delirium episode by reviewing the patient charts. We calculated the positive predictive value (PPV) with 95% confidence interval (95% CI) for all potential incident delirium episodes and for specific subgroups. Results: Within 10,515 rehabilitation stays we identified 554 potential incident delirium episodes. Overall, 125 potential incident delirium episodes were confirmed by expert review. The PPV of the chart-based method varied from 0.23 (95% CI 0.19–0.26) overall to 0.69 (95% CI 0.56–0.79) in specific subgroups. Conclusions: Our chart-based method was able to capture incident delirium episodes with low to moderate accuracy. By conducting an additional expert review of the medical charts, we identified a study population of validated incident delirium episodes. Our chart-based method contributes towards an automated detection of potential incident delirium episodes that, supplemented with expert review, efficiently yields a validated population of incident delirium episodes for research purposes.

## 1. Introduction

Delirium is an etiologically nonspecific organic cerebral syndrome characterized by concurrent disturbances of consciousness, attention, perception, thinking, memory, psychomotor behaviour, emotion, and the sleep–wake cycle [1,2]. Delirium can occur with or without pre-existing neurocognitive disorders and can vary in duration and severity [2].

Despite the fact that delirium has been associated with increased mortality in the rehabilitation setting as well as an increased risk for postsurgery and posthospital long-term consequences, to date, only few studies have assessed incidence and prevalence of delirium and associated risk factors in this setting [3,4,5,6,7,8,9,10]. This may primarily be explained by the lack of validated methods to identify delirium during rehabilitation. Consequently, the prevalence of delirium in electronic real-world databases, including rehabilitation databases which could be used to conduct observational studies, was reported to be severely underestimated [11,12].

Due to its highly fluctuating nature and several differential diagnoses with similar key symptoms, diagnosing delirium in inpatients is challenging [13]. Validated screening tools, such as the Confusion Assessment Method (CAM), have been developed to detect delirium in several inpatient settings [14,15,16]. However, as these tools are insufficiently validated in the rehabilitation setting, require specialized training and are time-consuming, standardized delirium screening in rehabilitation has remained rare [17]. Inouye et al. [18] proposed a different approach to identify potential delirium episodes based on systematic screening of inpatients’ medical charts, which was validated on a general medicine ward and subsequently used in several studies [4,19,20]. Another study compared the chart-based approach with the prospective interview-based screening instrument CAM, and suggested that the chart-based method was more likely to detect delirium episodes occurring outside the screening-times of interview-based methods, but less likely to detect hypoactive forms of delirium [21]. However, the key advantage of the chart-based method is its retrospective character, which allows the detection of potential delirium episodes by screening pre-existing clinical data.

Furthermore, Puelle et al. [22] published a list of delirium-predictive key words, which, combined with the method of Inouye, may serve as a starting point for developing an automated chart-based method to detect delirium episodes within a database of electronic medical records. A similar automated chart-based method to detect patients with dementia was already developed and validated [23].

ZURZACH Care is a Swiss group of hospitals and outpatient institutions specialized in inpatient and outpatient rehabilitation and prevention. Patient data have been recorded electronically since 2015 and comprise patient demographics, free-text medical notes, administered drugs, diagnoses and laboratory values. This data may be used to perform observational studies to better understand the incidence of delirium and related risk factors in the rehabilitation setting.

The first aim of this study was to develop an automated chart-based method to identify potentially incident delirium episodes within the electronic database of ZURZACH Care, based on the approach of identifying delirium-predictive key words in the medical charts of patients. Secondly, this study aimed to (i) validate this method by calculating the positive predictive value (PPV) of the chart-based method compared to confirmed incident delirium episodes and (ii) to compare the percentage of confirmed incident delirium episodes identified through expert review with the percentage of recorded delirium discharge diagnoses in the claims data. Thirdly, this study aimed to identify validated incident delirium episodes within the ZURZACH Care database for further research purposes, for example to gain insights into the clinical implications of delirium.

## 2. Materials and Methods

### 2.1. Data Source and Study Design

We conducted a validation study based on data derived from the electronic clinical database of ZURZACH Care between 2015 and 2018. Charts comprise patient demographics such as date of birth, sex and patient identification number, and inpatient care data such as case number (assigned per rehabilitation stay), rehabilitation program, and admission and discharge dates. During inpatient care, subjective and objective observations of nurses, physicians and therapists are documented as free-text medical notes, including date and time. Additionally, drug prescriptions including brand name, ATC-code, dosage, posology, time of prescription start, stop and administration are also documented [24]. At admission, all diagnoses deriving from the former care provider (e.g., acute hospital) are documented as free-text in the electronic clinical database, while at discharge, pre-existing and new diagnoses are coded within the ICD-10 classification system and archived as claims data [2].

### 2.2. Study Population

We included all patients who underwent ≥1 rehabilitation stay in angiology, cardiology, neurology, rheumatology, orthopaedics or headache or pain programs at ZURZACH Care, Rehaklinik Bad Zurzach, Switzerland, between 1 January 2015 and 31 December 2018. We considered each single rehabilitation stay of patients who were referred to inpatient rehabilitation several times during the study period.

### 2.3. Identification and Classification of Potential Incident Delirium Episodes

We performed the process to identify potential incident delirium episodes and to classify them in two steps: in a first step, experts performed a review on a sample of identified episodes. The knowledge gained in the sample review was implemented to improve the identification accuracy for the main review (second step).

#### 2.3.1. Sample Review

Within the study population, we identified all rehabilitation stays with ≥1 recorded delirium-predictive key word in the free-text medical notes (henceforth called “potential delirium episodes”). We defined delirium-predictive key words (henceforth abbreviated “key words”) as the German translation of any of the terms reported in the study of Puelle et al. [22], plus additional common terms used to describe patients experiencing delirium in the acute neurorehabilitation unit of ZURZACH Care. Additional terms were identified based on independent interviews with the head nurse of this unit and the medical director of neurology at ZURZACH Care. The resulting list of key words is provided in Table 1 (see Table S1 in the Supplementary Material for English translation).

We defined the date of the first recording of a key word during a potential delirium episode as the index date. As we intended to validate algorithms to capture incident delirium episodes during rehabilitation, we excluded all rehabilitation stays whose admission date was the same date as the index date, or whose free-text admission diagnoses comprised the terms for delirium used in German language (“Delir” or “Delirium”).

Among the identified potential incident delirium episodes, we randomly selected a sample of 100 episodes for review by two medical experts, a senior neurologist and a junior physician, both working in a neurological rehabilitation unit at ZURZACH Care. To achieve a standardized approach to the review process, we performed two specific training sessions with the experts, where they defined common evaluation strategies for the classification of potential delirium episodes.

For the review, profiles comprised the admission date and a chronological list of all medical notes registered at and after the index date. To limit the risk of observer bias by the experts due to identification of patients and recollection of associated medical events, we replaced the patient identification number and the case number by a neutral identification number in the profiles.

Based on clinical knowledge and predefined evaluation strategies, both physicians independently classified each potential incident delirium episode as “confirmed incident delirium episode”, “no incident delirium episode”, or “uncertain incident delirium episode”. If the classification was discordant between the two physicians, they had to find a verbal consensus (“confirmed incident delirium episode” or “no incident delirium episode”). Because our rehabilitation database lacks validated delirium screening results, and as the diagnosis of delirium in inpatients is often based on subjective clinical observations rather than on biological markers, we considered expert review to be the most accurate and feasible way to classify potential delirium episodes.

#### 2.3.2. Main Review

As a result of the sample review, we identified terms within the initial list of key words (Table 1) that were not specific enough for delirium detection, namely “unruh…” (German abbreviation for “restless”) because this term was often used to describe patients who were nervous or agitated for reasons other than delirium (i.e., argument with the roommate) or “orient…”, “kooperat…” (German abbreviations for “oriented” and “cooperative”) because these terms often referred to fully oriented and cooperative patients. Therefore, we excluded the term “unruh…” and we added a negation to the other two terms, i.e., substituted “orient…” by “nicht (…) orient…” (German abbreviation for “not oriented”) and “kooperat…” by “nicht (…) kooperat…” (German abbreviation for “not cooperative”). Table 1 provides the modified list of key words used for the main review (see Table S1 in the Supplementary Material for English translation). The sample review also demonstrated that the reviewers required ≥2 key words (instead of ≥1) to evaluate the characteristic fluctuation of delirium. Moreover, they required knowledge on antipsychotic, anxiolytic or hypnotic drugs (ATC-Codes: “N05Axxx”, “N05Bxxx”, “N05Cxxx”) prescribed within 12 h before, at, or at any time after the index date and a potential pre-existing diagnosis of dementia (ICD-10 Codes F00 to F03 incl. subgroups), because the differentiation between dementia, delirium and delirium superimposed on dementia or other psychoses is considered very challenging, and above-mentioned clinical data were required to distinguish between these diagnoses [25].

For the main review, we therefore identified all rehabilitation stays within the initial population with ≥2 key words as defined by the modified list of key words (Table 1). Within this revised population of potential delirium episodes, we defined the index date and excluded nonincident episodes as described under “Sample review” above.

Based on clinical knowledge and predefined evaluation strategies, the same two physicians who performed the sample review independently classified each potential incident delirium episode identified for the main review as “confirmed incident delirium episode”, “no incident delirium episode”, or “uncertain incident delirium episode”. If the classification was discordant, a second senior neurologist independently reviewed the concerned profiles. In these cases, if the classification of the first and the second senior neurologist was concordant, the classification of the junior physician was overruled. If the classification between the two senior neurologists was discordant, they had to discuss each single potential delirium episode until they found a verbal consensus.

### 2.4. Identification of Recorded Delirium Discharge Diagnoses within the Claims Data

Within the initial study population, we identified all rehabilitation stays with a recorded discharge diagnosis of delirium (ICD-10: “F05.xx”) within the claims data. The identified rehabilitation stays comprising a discharge diagnosis of delirium were then compared with the incident delirium episodes confirmed by the medical experts.

### 2.5. Statistical Analysis

We calculated the overall PPV with a 95% confidence interval (95% CI) of the described algorithm to identify incident delirium episodes during rehabilitation by dividing the number of (by expert review) confirmed delirium episodes (true positive) by the number of initially identified potential delirium episodes (true positive + false positive). Furthermore, we calculated PPVs with a 95% CI of different groups of identified potential delirium episodes according to the number of recorded key words (≥2; ≥3; ≥4; ≥5; ≥6; ≥7; ≥8; ≥9; ≥10) with or without the administration of ≥1 antipsychotic drug (ATC Codes N05Axxx) within 12 h before, at, or at any time after the index date and according to the rehabilitation discipline.

## 3. Results

**Figure 1:**
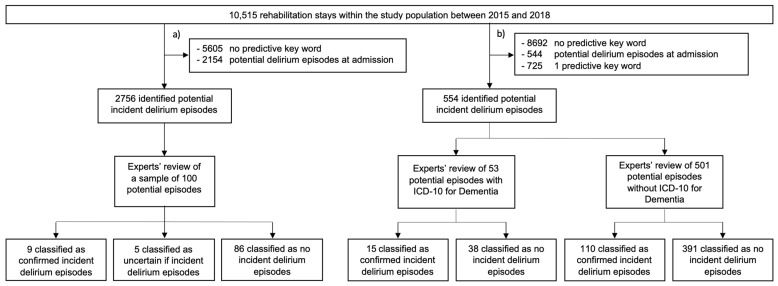
Selection and review process of potential incident delirium episodes within the initial population of rehabilitation stays: (**a**) Identification of patients based on the original delirium-predictive key words list for the sample review (Table 1); (**b**) Identification of patients based on the modified delirium-predictive key words list for the main review (Table 1).

Between 1 January 2015 and 31 December 2018, we identified 9406 patients who had a total of 10,515 rehabilitation stays. Baseline characteristics of the study population, median length of rehabilitation stay and rehabilitation disciplines are reported in Table 2. Within this population, we identified 4910 rehabilitation stays (46.7% of all stays) with ≥1 key word by applying the initial key words list (Table 1) for the sample review (the results of the sample review are illustrated in Figure 1a). By applying the modified key words list (Table 1) we identified 1823 rehabilitation stays (17.3% of all stays) for the main review. We excluded 314 rehabilitation stays because the index date corresponded to the admission date, 230 because the terms “Delir“ or “Delirium“ were comprised within the free-text admission diagnoses, and 725 because only one key word was recorded in the medical notes. This left us with 554 (5.3% of all stays) potential incident delirium episodes for experts’ review, 53 episodes (9.6%) of patients with dementia, and 501 (90.4%) without dementia (Figure 1b).

Overall, the two experts agreed on the classification of 405 episodes (93 classified as incident delirium episodes and 312 as no incident delirium episodes), and disagreed in the classification of 149 episodes (70 were classified as incident delirium episodes only by the senior neurologist, 69 were classified as incident delirium episodes only by the junior physician, and 10 were classified as uncertain by one of the two medical experts, and as no incident delirium episodes by the other) resulting in an agreement in 73.1% episodes. The patient profiles of the 149 episodes for which the two experts disagreed were reviewed by the second senior neurologist. For 49 episodes (32.9%), the two senior neurologists agreed on the classification and overruled the classification of the junior physician, while for 100 episodes (67.1%), the two senior neurologists had to reach a verbal consensus on the classification. Table 3 shows that 125 potential delirium episodes were classified as incident delirium episodes and 429 as no incident delirium episodes, resulting in a PPV of 0.23 (95% CI 0.19–0.26). Considering subjects with ≥6 recorded key words only, the PPV increased in those without or with ≥1 administered antipsychotic drugs within 12 h before, at, or at any time after the index date, to 0.55 (95% CI 0.46–0.64) and 0.69 (95% CI 0.56–0.79), respectively. Table 4 shows that both the PPV and the cumulative delirium incidence were highest among the rehabilitation discipline neurology, although differences in PPVs between rehabilitation disciplines and overall were small and statistically not significant.

Within the initially identified 10,515 rehabilitation stays, we identified 111 stays (1.1%) for which a discharge diagnosis of delirium was recorded in the claims data. Of these, 12 (10.8%) stays corresponded to an incident delirium episode confirmed by the medical experts.

## 4. Discussion

Our chart-based method was able to detect 554 potential incident delirium episodes within 10,515 rehabilitation stays (5.3%) in the ZURZACH Care database between 2015 to 2018. Among these, only 125 (1.2% of all stays, 22.6% of identified potential incident delirium episodes) episodes were confirmed as incident delirium episodes by expert review, resulting in a low-to-moderate accuracy of our chart-based method. The PPV of the method varied from 0.23 (95% CI 0.19–0.26) for potential episodes with ≥2 recorded delirium-predictive key words to 0.69 (95% CI 0.56–0.79) for potential episodes with ≥6 recorded key words and ≥1 recording of an administrated antipsychotic drug. The increase in the PPV was inversely related to the absolute number of identified incident delirium episodes. Considering only the rehabilitation discipline neurology, our method detected 343 (9.9% of all stays) potential incident delirium episodes. Among these, 89 (2.6% of all stays) episodes were confirmed as incident delirium episodes by expert review, resulting in PPV of 0.26 (95% CI 0.21–0.31). Both the proportion of detected potential incident delirium episodes and the PPV were higher in the neurology discipline than in non-neurological disciplines. However, it is important to emphasise that the PPV is dependent on the incidence or prevalence of a disease, and in this case, the incidence of delirium in neurology was about five times higher than in non-neurological disciplines. We found that for 1.1% of all rehabilitation stays, a discharge diagnosis of delirium was recorded in the claims data after the rehabilitation stay. Although this percentage seems similar to the proportion of confirmed incident delirium episodes in expert review (1.2%), the comparison of the single stays demonstrated a low concordance between the two groups. Only 10.8% of the identified rehabilitation stays with a discharge diagnosis of delirium corresponded to an incident delirium episode. Overall, we identified a study population of 125 validated delirium episodes.

The low-to-moderate accuracy of our chart-based method may be explained by the similar clinical manifestation of delirium with other neurological impairments due to pathologies such as stroke, status epilepticus, or dementia. These differential diagnoses result in the recording of similar keywords to delirium, and therefore have been captured as well by the chart-based method. Because such differential diagnoses are more common within neurologic rehabilitation, our thesis is supported by the higher proportion of ‘no incident delirium episodes’ (false positives) within this discipline (254 out of 3458 stays [7.3%]) compared to the other rehabilitation disciplines (175 out of 7057 [2.5%]). Differentiation between delirium episodes and differential diagnoses of delirium was only possible during the experts’ review process.

Depending on the clinical setting and the average age of the investigated populations, reported delirium incidence for non-intensive-care inpatients varies between 3% and 51% [11,26,27]. Considering these data, the 1.2% confirmed incident delirium episodes that we observed within all rehabilitation stays was lower than expected, but may be explained by considering the differences in studied populations and methodologies. We assessed the incidence of delirium in a rehabilitation setting across all age groups (around 50% of our study population was <70 years old), whereas most previous studies assessed the incidence of delirium only in elderly populations (>65 years old) and nonrehabilitation settings [11,12,27]. Additionally, most of the previous studies that were summarized in systematic reviews did not assess the patients’ history of delirium or assessed delirium symptoms at admission, which, due to the transient nature of delirium, may have led to inclusion of prevalent delirium episodes [11,27]. We placed emphasis on detecting only new episodes of delirium by excluding those stays already comprising a record of a delirium diagnosis or key words at admission date (5.2% of all rehabilitation stays). Finally, the large variation in the incidence of delirium reported in pre-existing literature is questionable, and highlights a considerable heterogeneity in the methodology used to assess delirium.

The low concordance between the rehabilitation stays with a discharge diagnosis of delirium recorded in the claims data and those with an incident delirium episode confirmed by the medical experts might have different reasons. First, some discharge diagnoses may originate from diagnoses made during the acute care hospitalisation prior to rehabilitation start. Second, unlike in the acute setting, where the focus is on diagnosis, the focus in rehabilitation is set on therapeutic aspects. Third, because reimbursement rates in Swiss rehabilitation are currently independent of new diagnoses made during rehabilitation, there is no direct financial interest to transfer new diagnoses, such as delirium, into the claims data. This result demonstrates that claims data are unsuitable to identify incident delirium episodes within such a database. This is compatible with a previous study, in which only 18% of all patients with assessed delirium, based on the prospective interview-based screening instrument CAM, also had a discharge diagnosis of delirium recorded in the claims data [28]. In addition, the percentage of rehabilitation stays with a discharge diagnosis of delirium recorded in the claims data was considerably lower than the delirium prevalence range reported in the literature, supporting the thesis that the prevalence of recorded delirium diagnoses in electronic real-world databases is severely underestimated [11,12].

We compared our results with those of Inouye et al. [18] who developed and prospectively validated a similar chart-based delirium detection method within 919 inpatients of a general medicine ward, achieving a PPV of 0.39 (95% CI 0.32–0.45) by comparing the chart-based method to the validated interview-based instrument CAM. In contrast to our method, the chart reviewers manually searched for potential delirium episodes, whereby they not only searched for delirium key terms but also for any evidence of “acute confusional state” present in all sections of the patient chart. In addition, they were able to calculate a sensitivity of 0.74 (95% CI 0.65–0.81) and a specificity of 0.83 (95% CI 0.80–0.86) of the chart-based method. Thus, although the sensitivity and specificity of their method was adequate, the PPV was not much higher than the overall PPV we calculated in our study, because of the low delirium prevalence in their study population. Because we did not review rehabilitation stays for which no key words were recorded in the medical notes (and therefore did not assess true negative or false negative episodes), we were not able to calculate the sensitivity and specificity of our method. Therefore, although the two studies had different aims, a different design and a different setting, the results of both studies demonstrate the limited suitability of clinical databases to detect delirium retrospectively, based exclusively on notes of evidence of confused state.

The following limitations of our study have to be mentioned. First, the detection of potential incident delirium episodes was based on identification of defined key words recorded in medical notes. The recording of medical notes is a nonstandardized procedure and is affected by interpersonal, interprofessional and interdisciplinary heterogeneity, as shown by the lower PPV within non-neurological disciplines. Therefore, in case of insufficient recording or use of nonconsidered key words, we could have missed some delirium episodes. We tried to limit this issue by reviewing medical notes of all rehabilitation staff including other specialists or therapists. Second, because the clinical manifestation of delirium is, as already mentioned, similar to other neurological impairments due to pathologies such as stroke, status epilepticus, or dementia, some key words were not specific enough to differentiate between delirium and its differential diagnoses. We attempted to improve the PPV of delirium diagnosis by considering only potential delirium episodes that were accompanied by records of antipsychotic drug administration shortly prior to or at any time after the first registered key word, as this class of drugs is often used to treat delirium in clinical practice. While this approach led to a moderate improvement in the PPV, it especially led to a loss of chart-review-confirmed incident delirium episodes for which no antipsychotics were prescribed. This indicates a nonspecific use of antipsychotic drugs in clinical practice, and that not every delirium episode is treated with antipsychotic drugs, but also with behavioural and environmental interventions. Third, because our method relies on the records of behavioural observations in medical notes, less noticeable episodes of hypoactive delirium could have been missed. Fourth, although we consider the review of at least two independent and specifically trained medical experts suitable to validate delirium episodes based on medical charts, their expertise remains subjective, as shown by the moderate concordance during the review process, which was around 73% and comparable with previous studies [29]. We tried to limit this subjectivity by involving a second senior neurologist who conducted an independent review and found a verbal consensus with the first senior neurologist on each single episode where the classification was discordant. Fifth and already mentioned, because expert review was based on the interpretation of key words in the clinical context, and therefore charts without key words were not reviewed, we were not able to determine the false negative episodes and thus could not calculate the sensitivity and specificity, nor the negative predictive value (NPV), of our method. However, based on the experience of past studies and on our effort to maximize delirium detection by adapting the initial list of key words, we can expect a limited number of false-negative delirium episodes [18]. Lastly, because we defined delirium-predictive key words in German language and completed the key words list with terms typically used to describe delirious patients in rehabilitation settings, the generalizability of our study findings is limited to the rehabilitation setting of German-speaking countries.

Our data suggest that retrospective detection of incident delirium episodes within routinely collected clinical data remains challenging. From our perspective, a chart-based delirium-detection method based on key words used to describe delirium symptoms can be useful to preselect potential delirium episodes for research purposes. It will thus reduce time-effort but will not replace expert profile review, which is expensive and not suitable for large databases. Our results are consistent with other studies, suggesting that strategies used to identify incident delirium in large clinical or claims databases by identifying recorded diagnoses are not sufficiently effective, because delirium is severely underdiagnosed in clinical practice [12,30]. There is a need to implement standardized delirium assessment and documentation methods during inpatient rehabilitation in order to improve the validity of delirium diagnoses within electronic databases. These standardized data would facilitate the investigation of delirium incidence and associated risk factors in rehabilitation, and thus have therapeutic implications.

## 5. Conclusions

Our chart-based method based on identifying delirium-predictive key words in the medical notes was able to detect incident delirium episodes within inpatients undertaking rehabilitation with low-to-moderate accuracy. Our chart-based method contributes towards an automated detection of potential incident delirium episodes that, supplemented with expert review, efficiently yields a validated population of incident delirium episodes for research purposes.

## Figures and Tables

**Table 1:** Lists of delirium-predictive key words.

(A)	(B)
agress *	agress *
aggress *	aggress *
delir *	delir *
desorient *	desorient *
durcheinand *	durcheinand *
halluzin *	halluzin *
klingelmatte	klingelmatte
konfus *	konfus *
unkoperat *	unkoperat *
unkooperat *	unkooperat *
nestel	nestel
orient *	nicht (…) orient *
koperat *	nicht (…) koperat *
kooperat *	nicht (…) kooperat *
unruh *	
verwirr *	verwirr *

(A) Key words derived from the literature and translated to German (**bold**) and further common German terms used to describe patients experiencing delirium. (B) Modified key words after the sample review. * indicates possible different endings. (…): any 0 to 12 characters.

**Table 2:** Baseline characteristics of the study population.

	Study Population (*n* = 10,515)
Male	4683 (44.54%)
Median length of stay in days (IQR)	22 (10)
Median age at admission in years (IQR)	70 (23)
Age at admission, years	
<40	700 (6.66%)
40–49	822 (7.82%)
50–59	1684 (16.02%)
60–69	1925 (18.31%)
70–79	2913 (27.70%)
80–89	2238 (21.28%)
>90	233 (2.22%)
Rehabilitation discipline	
Angiology	631 (6.00%)
Cardiology	1127 (10.72%)
Headache program	450 (4.28%)
Neurology	3458 (32.89%)
Orthopedics	2964 (28.19%)
Pain program	510 (4.85%)
Rheumatology	1095 (10.41%)
Others	280 (2.66%)

IQR: interquartile range.

**Table 3:** Number of potential and confirmed incident delirium episodes with positive predictive values (PPV) and 95% confidence intervals (95% CI) by number of recorded delirium-predictive key words and by administration of at least one antipsychotic drug within 12 h before, at, or at any time after the index date.

Number of Delirium-Predictive Key Words	≥1 Antipsychotic Drug after Index Date	Potential Incident Delirium Episodes	Classified Incident Delirium Episodes	PPV (95% CI)
≥2	No	554	125	0.23 (0.19–0.26)
	Yes	152	80	0.53 (0.45–0.61)
≥3	No	312	100	0.32 (0.27–0.37)
	Yes	110	63	0.57 (0.48–0.67)
≥4	No	197	85	0.43 (0.36–0.50)
	Yes	88	57	0.65 (0.55–0.75)
≥5	No	141	68	0.48 (0.40–0.57)
	Yes	68	46	0.68 (0.56–0.79)
≥6	No	105	58	0.55 (0.46–0.65)
	Yes	61	42	0.69 (0.57–0.81)
≥7	No	78	43	0.55 (0.44–0.66)
	Yes	51	33	0.65 (0.51–0.78)
≥8	No	61	33	0.54 (0.41–0.67)
	Yes	42	27	0.64 (0.49–0.79)
≥9	No	53	29	0.55 (0.41–0.69)
	Yes	38	24	0.63 (0.47–0.79)
≥10	No	42	22	0.52 (0.37–0.68)
	Yes	30	18	0.60 (0.41–0.79)

**Table 4:** Number of rehabilitation stays, potential and confirmed incident delirium episodes, cumulative delirium incidence and positive predictive values (PPV) with 95% confidence intervals (95% CI) by rehabilitation discipline and overall.

Rehabilitation Discipline	Rehabilitation Stays	Potential Incident Delirium Episodes	Classified Incident Delirium Episodes	Cumulative Delirium Incidence	PPV (95% CI)
Cardiology	1127	31	6	0.53%	0.19 (0.04–0.35)
Neurology	3458	343	89	2.57%	0.26 (0.21–0.31)
Orthopedics	2964	111	19	0.64%	0.17 (0.10–0.24)
Others **	2966	69	11	0.37%	0.16 (0.07–0.25)
	10,515	554	125	1.19%	0.23 (0.19–0.26)

**: Angiology, Headache program, Pain program, Rheumatology, others.

## Data Availability

Restrictions apply to the availability of these data. Data was obtained from the clinical database of ZURZACH Care and are available on request from the corresponding author with the permission of ZURZACH Care.

## Supplementary Materials

The following are available online at https://www.mdpi.com/article/10.3390/neurolint13040067/s1, Table S1: List of delirium-predictive key words translated in English.
