# Supplementary material for: Detecting Incident Delirium within Routinely Collected Inpatient Rehabilitation Data: Validation of a Chart-Based Method
Source: Neurol Int. 2021 Dec 9;13(4):701–11. doi: 10.3390/neurolint13040067 (PMC8705493; doi:10.3390/neurolint13040067)
Supplement: Supplementary file 1 [file neurolint-13-00067-s001.zip › neurolint-1392029-supplementary.pdf]

Supplementary Materials:

Table S1: Lists of delirium predictive key words.

A)	B)
agress*	agress*
aggress*	aggress*
**delir***	delir*
**disorient***	disorient*
jumbled	jumbled
**hallucin***	hallucin*
floor mat alarm	floor mat alarm
**confus***	confus*
uncoperat*	uncoperat*
uncooperat*	uncooperat*
nested	nested
**orient***	not (...) orient*
**coperat***	not (...) coperat*
**cooperat***	not (...) cooperat*
agitated	
addled	addled

A) Key words derived from the literature (**bold**) and further common terms used to describe patients experiencing delirium translated in English. B) Modified key words after the sample review. *: indicates possible different endings. (…): any 0 to 12 characters.
